# Editorial

**Published:** 1872-12

**Authors:** 


### EDITORIAL.

### Our Profession.

Recently our attention has been directed to the status of the profession in Ohio, rather more definitely than heretofore, and uro observation was made in one direction only. There was found much to encourage and stimulate in the way of practical and scientific advancement, and a more elevated position assumed, and broader views entertained, and more just appreciation of what the profession should be, and require of its members, and especially of those who are to enter its ranks in the future; yet there are some features that are discouraging, and of these we may mention the low degree of qualification possessed by so many of the profession in our state.

An illustration of this statement we have before us, a record of the number, ard sufficiently correct for our purpose, the professional status of the dentists of one of the populous and wealthy counties of our State: its people, intellectual, intelligent, and refined.

In that county there are twenty-seven dentists; of this number five are graduates of dental colleges—one of these of recent date; of the whole number not more than six or seven have had anything that could, by any sort of stretch, be called a dental pupilage; the others are almost wholly self-made dentists, and poorly made at that. Fourteen of the number have no established offices, but tinker-like travel from house to house, preying upon such victims as they can induce to submit to their mischievous practice. The chief work of such is to extract teeth, bad or good, to them it matters not which, and to insert artificial dentures, which are generally miserable caricatures of the natural teeth, they have so ruthlessly destroyed. One of these travelers displays a diploma from a dental college. Shame upon the man who will thus debase his profession, abuse the privileges he has had, bring disgrace upon his *Alma Mater*, scorn upon himself, and affliction and disappointment upon the people he professes to serve. Of the remaining thirteen about one-half of them in the practice of their profession serve those intrusted to their care, in good faith, and with decided success. Of the remaining ones of this number, though in established offices, we should be glad to have the evidence that would warrant the assertion that they do more good than evil.

Now this looks rather dark. Well, so it is; but we think there are but few counties in the State that will present such an exhibit; there are some no doubt, perhaps some worse, but we know there are many far better. And a very encouraging feature is that in Ohio this state of things is becoming rapidly changed.

The multiplication of incompetants and quacks, either by education or immigration has been most effectually checked for the last four years. Nearly all the empyrics in the State are old stagers, or at least have been here more than four years; and all of these within a very short time—January 1, 1873, must qualify themselves according law, leave the profession, or occupy a very uncomfortable and dangerous position

In many counties in the State we know there have been none practicing in violation of the law since its enactment; and we think that the instances in the State, where dentists have entered the practice contrary to the law, since its passage, have been very few indeed, and it is quite certain that no such one has much sympathy or encouragement from those who have or will conform to the law. Indeed those who conform to the requirement of the law can not in justice to themselves stand quietly by and see it violated.

### Close of the Volume.

This number closes the twenty-sixth volume of the DENTAL REGISTER. During the time of its existence dentistry has developed from a mere trade or handicraft to a recognized profession.

Twenty-five years ago the whole of dentistry was embodied in artistic skill. There were perhaps a few, and very few indeed, in our country, who had any proper comprehension or appreciation of what dental science ought to be, or even has now attained. And the facilities for the accomplishment of its work, even in an artistic aspect were so meagre and rude, that we can scarcely conceive how anything very valuable was attained.

But it is now fully conceded that dental mechanism, and facility of manipulation is really but the minor part of that which constitutes dental science and practice.

How much journalism has effected in the accomplishment of this work, others can judge as well as we; it has certainly not been one of the least instrumentalities in the work; whether the REGISTER has carried forward its share of the work, we will not now pretend to decide; it has done what it could.

This completes the eighteenth year of our editorial labor upon this work. It has involved unremitting toil, requiring almost daily attention during all these years.

The physical labor has been no small matter, and especially when the work of publishing, as well as editing, was upon our hands, which was the case about fourteen years of this time; in addition to the physical, some intellectual effort was expended.

And still further those only who have labored in such a field, can have any adequate idea of the responsibility that attaches to the work.

The impression or conviction that what is said, and given out to the profession, may be the occasion of much good or much evil, bears with it a sense of great responsibility, and this become intensified by the fact, that more is expected of one who attempts to conduct such an enterprise, than it is possible for any one man to possess.

We have labored in this direction for what we conceived to be for the best interests of the profession. Have received criticisms and castigations, submissively, and we trust profitably; have received commendations and expressions of warm appreciation, with thankfulness, and we trust without vanity. We have had hearty cooperation from some quarters in the profession, from others we have received no cooperation or assistance; for this however we make no complaint.

We shall in future, as heretofore, do the best we can, with the means placed at our disposal, for the devopment and progress of our chosen profession.

### Editorial Experiences.

He who starts out in journalism with the intention of pleasing every body, will very soon become aware of his inability to carry out his intention. He may select his matter as well as possible from what is presented, and spend much time in its preparation, yet there will be some to criticise and find fault, object to the matter or the manner. One says the articles are too abstruse—too scientific; another that they are too practical and do not deal enough in principles. One says they are too long and too heavy, another says they are short and frivolous; one says "why did you publish that article of A's, on 'Miscellanies.' "If the REGISTER is to be made up of that kind of stuff we don't want it." Another says, "Those Miscellanies of A's are about the best and most instructive I have ever seen." Another says, "Why hasn't the REGISTER more editorials?" that it contains a few short bits of editorials that amount to but little." Another says, "THE REGISTER contains more editorial than any other two dental journals in the country, and they are pointed and practical."

Now we suppose that every dental journal in the country has its share of these encouragements.

The Editor is somewhat like the ten pins in the alley, one sets him up, and another knocks him down; and happy is he that can feel when he is knocked down that he is to be set up again.

An editor can not and should not be held responsible for what he publishes in the original department of his journal. It is oftentimes his duty to publish that with which he does not agree. Every rational and reasonable subject pertaining to the profession should have a hearing. He who attempts to force every one's ideas into his own mould, will fall far short of his duty. It is sometimes well to present false and erroneous ideas and practices, that they may be criticised and their errors corrected. Many wrong views are entertained, and modes of practice employed that need only the full clear light of investigation to be corrected—truth need never fear in equal combat with error.

### A Word to Contributors.

We have occasionally suggested that some who contribute to the Register do not prepare their manuscript as well as they should. Many write hastily and carelessly, and then request that the matter be put in good shape before being printed. This may be well enough for the writers, if there chirography can be deciphered, which is sometimes not an easy matter; but it is not so well for the editor. We have often spent more time and labor in correcting and reconstructing articles than it required to write them. Not only in chirography but in orthography, punctuation, and the structure of language are there very great defects with many writers. Now all can remedy these defects if they will.

Our request is, write plainly, and especially let all unusal words and proper names be most distinctly written; and since expression is very much modified and governed by punctuation, it should receive strict attention by every writer.

Illegible writing is a fault for which their is no valid excuse; but proficiency in spelling, punctuation, and structure of language is only attainable, by persevering industry in the way of study and investigation; but it is an industry that brings a glorious reward

We shouid sometimes be discouraged were it not that so far as the preparation of manuscript, is concerned the Register has some of the best contributors in the country.

### A Card from Johnston Bros.

Owing to the difficulty found in keeping the "Back-action attachment" of the Morriton Engine in order, we have now discontinued making it or furnishing it with the Engine. We have now nearly completed a new hand piece, fitted to prevent the burs from slipping from the tool-holder, or from being loosened therefrom while in use. It will be put on all Morrison Engines made hereafter. This will be advertised in the next number of this journal.

JOHNSTON BROS.

